# Associations of Long-Term Physical Activity Trajectories With All-Cause Mortality in a General Population

**DOI:** 10.3389/ijph.2023.1605332

**Published:** 2023-01-16

**Authors:** Chia-Lin Lee, Wei-Ju Liu, Ching-Hsien Chen, Jun-Sing Wang

**Affiliations:** ^1^ Division of Endocrinology and Metabolism, Department of Internal Medicine, Taichung Veterans General Hospital, Taichung, Taiwan; ^2^ Department of Medicine, School of Medicine, National Yang Ming Chiao Tung University, Taipei, Taiwan; ^3^ Department of Post-Baccalaureate Medicine, College of Medicine, National Chung Hsing University, Taichung, Taiwan; ^4^ Intelligent Data Mining Laboratory, Department of Medical Research, Taichung Veterans General Hospital, Taichung, Taiwan; ^5^ Divisions of Nephrology and Pulmonary, Critical Care and Sleep Medicine, Department of Internal Medicine, University of California, Davis, Davis, CA, United States; ^6^ Rong Hsing Research Center for Translational Medicine, Institute of Biomedical Science, National Chung Hsing University, Taichung, Taiwan

**Keywords:** mortality, trajectory, physical activity, metabolic equivalent, population

## Abstract

**Objectives:** We investigated the associations of mean levels of leisure-time physical activity (LTPA) and latent LTPA trajectories with all-cause mortality risk.

**Methods:** Trajectories of LTPA were established using group-based trajectory analysis with a latent class growth model in a population-based cohort between 1996 and 2014. A Cox-proportional hazard model was conducted to examine the associations of LTPA quintiles and LTPA trajectories with all-cause mortality.

**Results:** A total of 21,211 participants (age 18–90 years) were analyzed (median follow-up 16.8 years). The study participants were divided into five groups according to percentiles of LTPA (<20th, 20th–<40th, 40th–<60th, 60th–<80th, ≥80th) and LTPA trajectories (low/stable, medium/stable, increasing, decreasing, and fluctuating), respectively. Participants with a decreasing trajectory did not have a significantly lower risk of all-cause mortality despite having the highest baseline level of LTPA. In contrast, participants with a medium/stable (HR 0.84, 95% CI 0.72–0.98, *p* = 0.031) or an increasing (HR 0.57, 95% CI 0.33–0.97, *p* = 0.037) trajectory had a significantly lower risk of all-cause mortality.

**Conclusion:** Promotion of maintaining stable LTPA is beneficial for public health and survival.

## Introduction

Adopting a healthy lifestyle has been associated with longevity [1]. Physical activity is an important part of healthy lifestyles [1, 2], whereas physical inactivity has been shown to be associated with various non-communicable diseases, which are major burdens of public health worldwide [3]. Unfortunately, levels of physical activity have declined in most developed countries in recent decades [4, 5]. This decline may have contributed, at least in part, to the decrease in life expectancy [3–6]. According to a previous report [3], physical inactivity accounted for 9% of premature mortality worldwide in 2008. The authors estimated that if inactivity were decreased by 10%, more than 533 thousand deaths could be averted every year [3].

Evidence supporting the importance of maintaining physical activity to promote health has been found in a number of studies, which have reported an association of leisure-time physical activity (LTPA) with risk of mortality [7–13]. Nevertheless, the assessment of physical activity was conducted only at baseline in most of these investigations [9–13]. As lifestyle and physical activity might change during the follow-up period, this could constitute an important confounding factor when investigating the association between baseline LTPA and risk of all-cause mortality. To address this issue, levels of physical activity were assessed at several time points (mostly at two time points) in some studies [7, 8, 14–16] investigating their effects on risk of mortality.

In this study, we collected data on LTPA at several time points in a general population to establish LTPA trajectories using a validated method [17, 18] and investigated the associations of mean levels of LTPA and LTPA trajectories with risk of all-cause and cardiovascular/cancer mortality. The joint effects of LTPA levels and LTPA trajectories on all-cause and cardiovascular/cancer mortality were also examined.

## Methods

This study was conducted in accordance with the Declaration of Helsinki. We analyzed data from a population-based cohort established by the MJ Health Management Institution, Taipei, Taiwan. All participants had undergone a health check-up between 1996 and 2014, and provided informed consent to authorize the MJ Health Management Institution to process data for research use. To apply the data, we firstly had our study protocol approved by an Institutional Review Board. We then applied for data from the MJ Health Database/MJ Health Survey Data with the approval of our study protocol. The MJ Health Management Institution approved our application and we have the data for analyses. The check-ups of each participant included anthropometric measurements, physical examination, laboratory tests (including fasting plasma glucose, serum creatinine, lipids profile, …), and questionnaires on medical history and assessment of LTPA. We selected participants who were at least 18 years old and had received more than 5 health check-ups in an 8-year period between 1996 and 2014 to establish their trajectories of LTPA for analyses.

**FIGURE 1:**
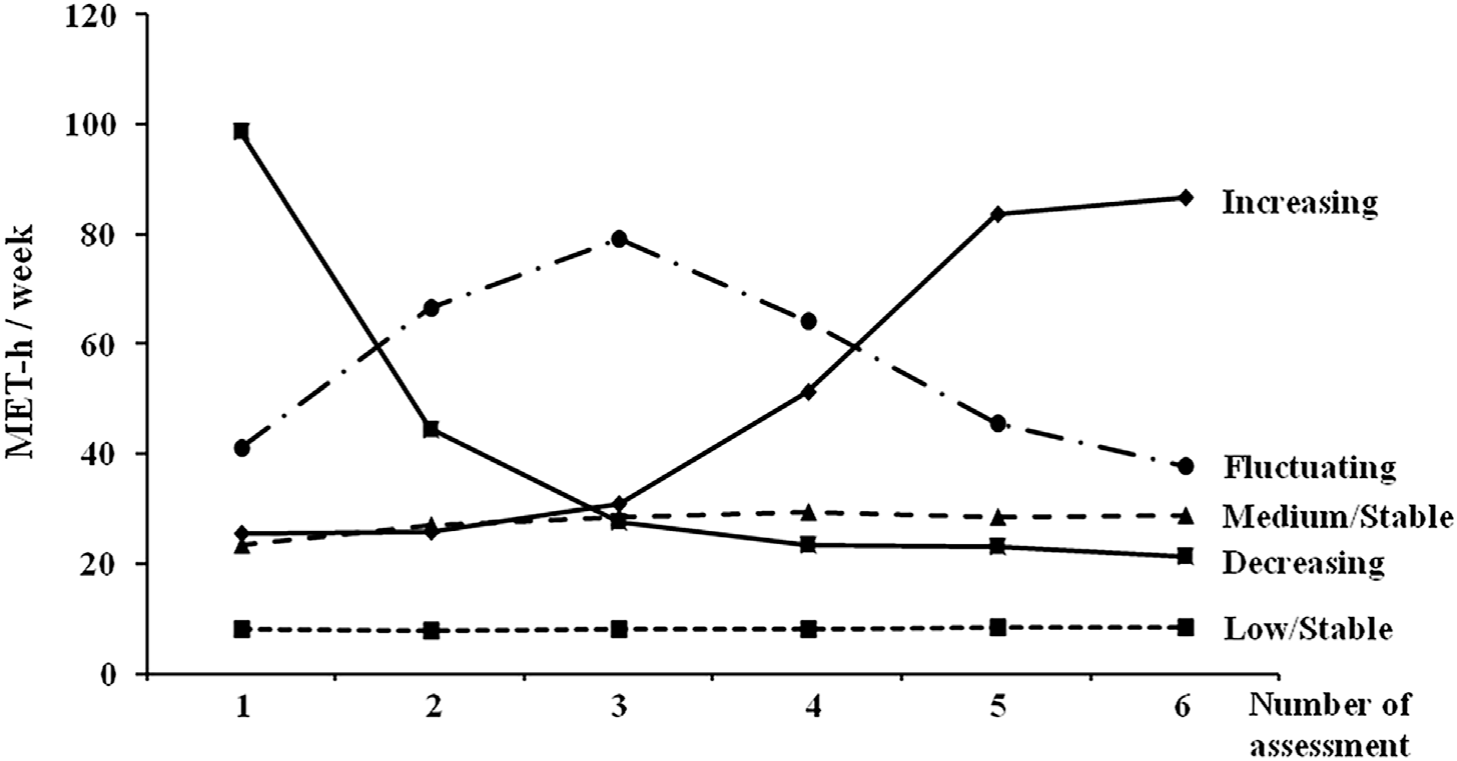
Latent trajectories of leisure-time physical activity levels (MJ cohort, Taiwan, 1996–2014). The time interval between each assessment was at least 1 year. MET, metabolic equivalent.

Using information from the validated questionnaires [9, 19, 20], we determined the intensity, duration, and frequency of LTPA for each participant. The intensity was assessed using metabolic equivalent (MET) [9, 21]. A MET value was assigned [21] according to the intensity of physical activity (2.5 for light, 4.5 for moderate, 6.5 for medium-vigorous, and 8.5 for high-vigorous exercise). The MET value was multiplied by duration and frequency, and was expressed as MET-h per week. Repeated measures of the MET value were used to establish trajectories of LTPA (MET-h per week) using group-based trajectory analysis with a latent class growth model [17, 18]. We decided the number of trajectories according to the Information of Bayesian Information Criterion (BIC) [22]. Then the study participants were assigned to distinct trajectory groups by posterior probabilities from the trajectory model. This method allowed us to group study participants by “naturally occurring” LTPA trajectories [23]. The use of a censored normal model was appropriate due to the continuous outcomes of MET. The models were fitted using the SAS ProcTraj procedure [22]. Finally, five trajectories of LTPA were established (Figure 1; Supplementary Table S1).

The study participants were divided into five groups according to percentiles (<20th, 20th–<40th, 40th–<60th, 60th–<80th, ≥80th) of their mean MET-h per week and their trajectories of LTPA (low/stable, medium/stable, increasing, decreasing, and fluctuating, Figure 1), respectively. The primary outcome was all-cause mortality. Data on mortality up to May 2021 were obtained. The associations of quintiles of LTPA and LTPA trajectories with all-cause and cause specific (cardiovascular and cancer) mortality were examined.

All of the statistical analyses were conducted using SAS software (Version 9.4; SAS Institute, Inc., Cary, NC, United States). The statistical significance of the differences in categorical and continuous variables among the LTPA percentiles and LTPA trajectory groups were examined using the Chi-square test and one-way ANOVA, respectively. To examine the associations of LTPA quintiles and LTPA trajectories with all-cause mortality, a Cox-proportional hazard model was used with adjustments for age, sex, body mass index, systolic blood pressure, smoking, history of cardiovascular disease, fasting plasma glucose, total cholesterol, and renal function. For cause-specific mortality, we considered cardiovascular death a competing event for cancer death, and *vice versa*. We conducted an extended Cox’s proportional hazards model to determine the subdistribution hazard ratio (SHR) [24] of cardiovascular and cancer mortality among the quintiles of LTPA and LTPA trajectory groups. To examine the joint effects of mean levels of LTPA and LTPA trajectories on all-cause and cause-specific mortality, we regrouped the study population according to quintiles of LTPA and LTPA trajectories. In all of the analyses, a two-sided *p*-value of <0.05 was considered statistically significant.

## Results

A total of 21,211 participants were analyzed, and the median follow-up time was 16.8 years. Table 1 shows the baseline characteristics of the study participants according to percentile of their mean MET-h per week during the study period. Participants in the highest quintile were older and more likely to be male, had a higher body mass index and blood pressure, and had a worse lipid profile (higher total and low-density lipoprotein cholesterol and triglyceride, and lower high-density lipoprotein cholesterol) than those in the lower quintiles.

**TABLE 1:** Baseline characteristics of the study participants according to percentile of mean physical activity levels (MJ cohort, Taiwan, 1996–2014).

Variable (number of participants with miss data)	Percentile of mean MET-h per week					*p*-value
	20th percentile	20th–40th percentile	40th–60th percentile	60th–80th percentile	≥80th percentile	
N	4242	4233	4249	4244	4243	
MET-h, per week	1.84 ± 3.17	5.67 ± 5.93	10.02 ± 8.94	16.05 ± 12.93	32.63 ± 27.48	<0.001
Age, years	34.8 ± 9.1	36.4 ± 10.4	38.3 ± 11.4	41.6 ± 13.4	43.9 ± 14.3	<0.001
Male, n (%)	1930 (45.5)	2134 (50.4)	2395 (56.4)	2537 (59.8)	2767 (65.2)	<0.001
Body mass index, kg/m^2^ (3)	22.5 ± 3.7	22.8 ± 3.5	23.1 ± 3.4	23.3 ± 3.3	23.5 ± 3.3	<0.001
Systolic BP, mm Hg (635)	115 ± 15	117 ± 16	119 ± 16	121 ± 17	123 ± 18	<0.001
Diastolic BP, mm Hg (635)	69 ± 11	70 ± 11	72 ± 11	73 ± 11	74 ± 12	<0.001
Smoking, n (%) (1495)	984 (24.3)	915 (23.1)	886 (22.5)	873 (22.5)	859 (22.1)	0.136
History of CVD, n (%) (1)	48 (1.1)	51 (1.2)	67 (1.6)	72 (1.7)	115 (2.7)	<0.001
FPG, mmol/L (25)	5.36 ± 0.92	5.39 ± 0.90	5.45 ± 0.92	5.48 ± 0.97	5.54 ± 1.01	<0.001
eGFR, ml/min/1.73 m^2^ (9)	88.8 ± 16.2	87.2 ± 15.7	85.8 ± 18.0	84.1 ± 18.4	83.2 ± 21.3	<0.001
Total cholesterol, mmol/L (22)	4.90 ± 0.88	4.92 ± 0.87	4.99 ± 0.92	5.06 ± 0.94	5.09 ± 0.95	<0.001
LDL cholesterol, mmol/L (1200)	2.90 ± 0.79	2.96 ± 0.78	3.02 ± 0.82	3.10 ± 0.85	3.13 ± 0.86	<0.001
HDL cholesterol, mmol/L (1125)	1.46 ± 0.40	1.42 ± 0.39	1.40 ± 0.39	1.38 ± 0.39	1.38 ± 0.38	<0.001
Triglyceride, mmol/L (11)	1.22 ± 1.04	1.24 ± 0.97	1.28 ± 1.02	1.34 ± 1.05	1.30 ± 0.97	<0.001

Data are presented as mean (95% CI) or n (%). CVD, cardiovascular disease; BP, blood pressure; eGFR, estimated glomerular filtration rate; FPG, fasting plasma glucose; HDL, high-density lipoprotein; LDL, low-density lipoprotein; MET, metabolic equivalent.

Table 2 shows the baseline characteristics according to the study participants’ latent trajectory of LTPA during the study period. Participants with a low/stable trajectory had the lowest mean MET-h per week (8.01 ± 9.60), while those with a decreasing trajectory had the highest mean MET-h per week (98.77 ± 33.99). The two groups were younger, had a lower body mass index and blood pressure, and had a better lipid profile (lower total and low-density lipoprotein cholesterol and triglyceride, and higher high-density lipoprotein cholesterol) than the other three groups.

**TABLE 2:** Baseline characteristics of the study participants according to latent trajectory of physical activity levels (MJ cohort, Taiwan, 1996–2014).

Variable	Latent trajectory of physical activity levels					*p*-value
	Low/Stable	Medium/Stable	Increasing	Decreasing	Fluctuating	
N (%)	16,100 (75.9%)	3913 (18.4%)	436 (2.1%)	294 (1.4%)	468 (2.2%)	
MET-h, per week	8.01 ± 9.60	23.38 ± 15.87	25.53 ± 21.24	98.77 ± 33.99	41.00 ± 26.62	<0.001
Age, years	37.5 ± 11.2	44.2 ± 14.1	42.8 ± 14.2	38.4 ± 14.3	44.8 ± 14.0	<0.001
Male, n (%)	8469 (52.6)	2447 (62.5)	301 (69.0)	208 (70.8)	338 (72.2)	<0.001
Body mass index, kg/m^2^	22.9 ± 3.5	23.5 ± 3.2	23.7 ± 3.7	23.0 ± 3.4	23.6 ± 3.1	<0.001
Systolic BP, mm Hg	118 ± 16	123 ± 18	122 ± 16	121 ± 17	123 ± 18	<0.001
Diastolic BP, mm Hg	71 ± 11	74 ± 11	73 ± 12	73 ± 11	74 ± 12	<0.001
Smoking, n (%)	3481 (23.2)	779 (21.8)	102 (25.5)	63 (23.3)	92 (21.3)	0.266
History of CVD, n (%)	224 (1.4)	96 (2.5)	10 (2.3)	8 (2.7)	15 (3.2)	<0.001
FPG, mmol/L	5.42 ± 0.93	5.54 ± 0.98	5.57 ± 0.89	5.5 ± 0.98	5.57 ± 1.26	<0.001
eGFR, ml/min/1.73 m^2^	86.6 ± 17.1	82.9 ± 19.9	86.9 ± 29.7	87.0 ± 22.5	80.5 ± 18.7	<0.001
Total cholesterol, mmol/L	4.96 ± 0.91	5.11 ± 0.94	5.08 ± 0.91	4.94 ± 0.98	5.08 ± 0.94	<0.001
LDL cholesterol, mmol/L	2.99 ± 0.81	3.15 ± 0.85	3.11 ± 0.85	2.99 ± 0.93	3.07 ± 0.81	<0.001
HDL cholesterol, mmol/L	1.41 ± 0.40	1.37 ± 0.38	1.40 ± 0.39	1.48 ± 0.36	1.41 ± 0.41	<0.001
Triglyceride, mmol/L	1.27 ± 1.03	1.33 ± 0.98	1.28 ± 0.78	1.06 ± 0.61	1.34 ± 1.20	<0.001

Data are presented as mean (95% CI) or n (%). CVD, cardiovascular disease; BP, blood pressure; eGFR, estimated glomerular filtration rate; FPG, fasting plasma glucose; HDL, high-density lipoprotein; LDL, low-density lipoprotein; MET, metabolic equivalent.

During the follow-up period, a total of 960 participants died (morality rate 264.9 per 100,000 person-years). The numbers for cardiovascular and cancer death were 177 and 399, respectively. The associations of baseline characteristics and physical activity levels with all-cause mortality are shown in Supplementary Table S2. Table 3 shows the associations of quintiles of LTPA and distinct LTPA trajectories with all-cause and cardiovascular/cancer mortality. Compared with participants who had the lowest quintile (<20th percentile), only those with the highest quintile (≥80th percentile) had a significantly lower risk of all-cause mortality (HR 0.76, 95% CI 0.58–0.99, *p* = 0.041) after multivariate adjustment. With regard to LTPA trajectories (using the low/stable trajectory as the reference group), participants with a decreasing trajectory did not have a significantly lower risk of all-cause mortality (HR 0.58, 95% CI 0.27–1.22, *p* = 0.151) despite having the highest mean MET-h per week. In contrast, participants with a medium/stable (HR 0.84, 95% CI 0.72–0.98, *p* = 0.031) or an increasing (HR 0.57, 95% CI 0.33–0.97, *p* = 0.037) trajectory had a significantly lower risk of all-cause mortality, compared with the reference group. Similar findings were noted with respect to cancer mortality.

**TABLE 3:** Cox proportional hazards models for all-cause and cardiovascular/cancer mortality by percentile of mean physical activity levels and latent trajectory groups (MJ cohort, Taiwan, 1996–2014).

	All-cause mortality				Cardiovascular mortality		Cancer mortality	
	Univariate	*p*	Multivariate^a^	*p*	Multivariate^b^	*p*	Multivariate^b^	*p*
	HR (95% CI)		HR (95% CI)		SHR (95% CI)		SHR (95% CI)	
Percentile								
<20th	1 (reference)		1 (reference)		1 (reference)		1 (reference)	
20th–<40th	1.10 (0.83–1.46)	0.495	0.84 (0.62–1.15)	0.271	1.39 (0.63–3.06)	0.410	0.79 (0.50–1.23)	0.291
40th–<60th	1.48 (1.14–1.92)	0.003	0.96 (0.72–1.27)	0.760	1.01 (0.47–2.17)	0.979	1.18 (0.79–1.74)	0.419
60th–<80th	2.12 (1.66–2.71)	<0.001	0.84 (0.64–1.10)	0.198	0.98 (0.47–2.03)	0.957	0.88 (0.59–1.30)	0.523
≥80th	2.51 (1.97–3.19)	<0.001	0.76 (0.58–0.99)	0.041	1.15 (0.57–2.33)	0.703	0.69 (0.46–1.04)	0.078
Trajectory								
Low/Stable	1 (reference)		1 (reference)		1 (reference)		1 (reference)	
Medium/Stable	1.76 (1.53–2.02)	<0.001	0.84 (0.72–0.98)	0.031	1.03 (0.72–1.48)	0.854	0.68 (0.52–0.89)	0.005
Increasing	1.14 (0.72–1.79)	0.588	0.57 (0.33–0.97)	0.037	0.82 (0.26–2.54)	0.729	0.71 (0.34–1.51)	0.377
Decreasing	0.65 (0.31–1.38)	0.262	0.58 (0.27–1.22)	0.151	1.48 (0.47–4.62)	0.501	0.40 (0.10–1.65)	0.206
Fluctuating	2.07 (1.51–2.83)	<0.001	0.99 (0.70–1.39)	0.947	1.35 (0.68–2.67)	0.394	1.05 (0.62–1.77)	0.852

^a^ Adjusted for age, sex, body mass index, systolic blood pressure, smoking, history of cardiovascular disease, fasting plasma glucose, estimated glomerular filtration rate, and total cholesterol.

^b^ Considering competing risk of mortality in addition to multivariate adjustment. MET, metabolic equivalent; SHR, subdistribution hazard ratio.

Table 4 shows the distribution of study participants by quintiles of LTPA and LTPA trajectories. It is interesting to note that participants with an increasing (group 7), decreasing (group 8), and fluctuating (group 9) trajectory had a mean LTPA level in the highest quintile (≥80th percentile). Nevertheless, their risks of all-cause mortality might be different. Using group 1 as the reference group, the joint effects of mean levels of LTPA and LTPA trajectories on all-cause and cardiovascular/cancer mortality are shown in Table 5. After multivariate adjustment, group 7 (≥80th percentile of LTPA with an increasing trajectory) had a significantly lower risk of all-cause mortality (HR 0.52, 95% CI 0.29–0.92, *p* = 0.024). A similar finding (HR 0.77, 95% CI 0.59–1.02, *p* = 0.066) was noted for group 6 (≥80th percentile of LTPA with a medium/stable trajectory).

**TABLE 4:** Distribution of study participants by percentile of mean physical activity levels and latent trajectory groups (MJ cohort, Taiwan, 1996–2014).

Percentile of mean MET-h	Latent trajectory of physical activity levels					Total
	Low/Stable	Medium/Stable	Increasing	Decreasing	Fluctuating	
<20th	4242 (20.0) (Group 1)	0	0	0	0	4,242 (20.0)
20th–<40th	4233 (20.0) (Group 2)	0	0	0	0	4,233 (20.0)
40th–<60th	4249 (20.0) (Group 3)	0	0	0	0	4,249 (20.0)
60th–<80th	3376 (15.9) (Group 4)	854 (4.0) (Group 5)	1 (0.0)	13 (0.1)	0	4,244 (20.0)
≥80th	0	3059 (14.4) (Group 6)	435 (2.1) (Group 7)	281 (1.3) (Group 8)	468 (2.2) (Group 9)	4,243 (20.0)
Total	16,100 (75.9)	3913 (18.5)	436 (2.1)	294 (1.4)	468 (2.2)	21,211 (100)

Data are presented as n (%). MET, metabolic equivalents.

**TABLE 5:** Joint effects of mean physical activity level and physical activity trajectory on all-cause and cardiovascular/cancer mortality (MJ cohort, Taiwan, 1996–2014).

Groups	All-cause mortality				Cardiovascular mortality		Cancer mortality	
	Univariate	*p*	Multivariate^a^	*P*	Multivariate^b^	*p*	Multivariate^b^	*p*
	HR (95% CI)		HR (95% CI)		SHR (95% CI)		SHR (95% CI)	
1: <20th percentile; Low/Stable	1 (reference)		1 (reference)		1 (reference)		1 (reference)	
2: 20th–<40th percentile; Low/Stable	1.10 (0.83–1.46)	0.491	0.84 (0.62–1.15)	0.274	1.39 (0.64–3.06)	0.408	0.79 (0.50–1.23)	0.293
3: 40th–<60th percentile; Low/Stable	1.48 (1.14–1.92)	0.003	0.96 (0.73–1.27)	0.769	1.01 (0.47–2.17)	0.977	1.18 (0.79–1.74)	0.417
4: 60th–<80th percentile; Low/Stable	2.13 (1.65–2.74)	<0.001	0.87 (0.66–1.15)	0.332	0.97 (0.46–2.05)	0.942	0.93 (0.62–1.39)	0.707
5: 60th–<80th percentile; Medium/Stable	2.13 (1.52–3.00)	<0.001	0.73 (0.50–1.07)	0.104	1.01 (0.39–2.60)	0.990	0.73 (0.41–1.30)	0.283
6: ≥80th percentile; Medium/Stable	2.66 (2.08–3.40)	<0.001	0.77 (0.59–1.02)	0.066	1.11 (0.54–2.30)	0.771	0.66 (0.43–1.01)	0.055
7: ≥80th percentile; Increasing	1.64 (1.00–2.70)	0.051	0.52 (0.29–0.92)	0.024	0.87 (0.24–3.17)	0.835	0.70 (0.31–1.58)	0.389
8: ≥80th percentile; Decreasing	0.98 (0.45–2.11)	0.954	0.53 (0.24–1.16)	0.111	1.58 (0.43–5.78)	0.491	0.40 (0.09–1.72)	0.218
9: ≥80th percentile; Fluctuating	3.00 (2.07–4.35)	<0.001	0.90 (0.60–1.35)	0.602	1.43 (0.57–3.59)	0.442	1.03 (0.56–1.90)	0.917

^a^ Adjusted for age, sex, body mass index, systolic blood pressure, smoking, history of cardiovascular disease, fasting plasma glucose, estimated glomerular filtration rate, and total cholesterol.

^b^ Considering competing risk of mortality in addition to multivariate adjustment. MET, metabolic equivalent; SHR, subdistribution hazard ratio.

## Discussion

In this study on a large general population with long-term follow-up, we demonstrated that LTPA trajectories (medium/stable or increasing) were associated with a decrease in all-cause and cancer mortality (Table 3). Although the study participants with LTPA ≥80th percentile had a significantly lower risk of all-cause mortality with a modest decrease in cancer mortality (Table 3), the benefit in survival was only observed in those with a medium/stable (group 6) or increasing (group 7) trajectory of LTPA (Table 5). Our findings highlight the importance of maintaining a stable amount of physical activity, in terms of survival in a general population.

Maintaining a physically active lifestyle has been associated with a lower risk of all-cause mortality. In contrast to most previous studies [7-16, 25] in which levels of physical activity were assessed only at baseline or at two time points, we used several assessments of LTPA to establish latent trajectories of physical activity levels during the study period. This method allowed us to categorize the study population into several groups according to naturally occurring trajectories [23, 26-28] rather than artificially defined cutoff values. Our findings were consistent with previous results [7-16, 25-29] that showed maintaining (medium/stable) or increasing physical activity levels was associated with a lower risk of all-cause mortality. In contrast, those who had decreasing or fluctuating LTPA trajectory did not have a significantly lower risk of mortality (Table 3) despite having a higher mean MET-h per week (Table 2) during the study period. These results indicate that LTPA trajectory is important with regard to the benefit in survival [1, 2] in the general population.

We further examined the joint effects of mean levels of LTPA and LTPA trajectories on all-cause mortality. Although participants who had ≥80th percentile of LTPA in our study had a lower risk of all-cause mortality (adjusted HR 0.76, 95% CI 0.58–0.99, *p* = 0.041, Table 3), the benefit was only observed in those who also had an increasing LTPA trajectory (group 7 in Tables 4, 5). Group 5, group 6, and group 8 had a modest, but non-significant, decrease in all-cause mortality risk compared with the least active group (group 1, Tables 4, 5). It is interesting to note that group 8 (281 of 294 participants with a decreasing LTPA trajectory) had a mean MET-h per week of about 100 (Table 2). The values in group 5/group6 and group 7 were around 23 and 25, respectively. These findings support the notion that maintaining an active lifestyle confers a survival advantage. Similar to our findings, a previous cohort study reported that a change from being active at baseline to being sedentary during follow-up in women aged 65 years or older was associated with a similar risk of all-cause mortality compared with those who stayed sedentary [7].

The decrease of all-cause mortality risk in the medium/stable and increasing LTPA trajectory was likely due to a lower risk of cancer mortality, rather than cardiovascular mortality (Table 3). This finding was similar to some previous studies [30-32], although there were inconsistent results [33-35]. In the aforementioned studies [30-35], cancer mortality was not considered a competing event for cardiovascular mortality (and *vice versa*) [24]. This may help explain the inconsistent findings. Since cancer and cardiovascular diseases share common risk factors (such as obesity, sedentary lifestyle, diabetes) [36], it is not surprising that maintaining physical activity was associated with a lower risk of either cardiovascular or cancer mortality. We speculate that the non-significantly lower risk of cardiovascular and cancer mortality in our study participants with an increasing LTPA trajectory might be due to the relatively small sample size. The decrease in cancer mortality risk in the study population with a medium/stable LTPA trajectory supports previous findings [30-32], and underlines the importance of adopting a healthy lifestyle in reducing cancer mortality [37], which has become one of the main causes of death worldwide [38].

This study has some limitations. First, this was a cohort study rather than a randomized controlled trial. Although we adjusted for multiple relevant variables in the statistical models, we cannot exclude the possibility that our findings were confounded by between-group differences in baseline characteristics. Nevertheless, long-term follow-up is needed to investigate the effects of physical activity on mortality risk. There would likely be considerable variation among study participants in the persistence of physical activity over time. Hence, randomizing study participants to different levels of physical activity for a long period of time might be impractical. As the persistence of physical activity is important in terms of its effects on outcomes [14-16, 25-29], our method of establishing latent physical activity trajectories [17-18, 23] could help address this issue despite its limitations. Second, data on LTPA were collected through self-reported questionnaires. We analyzed data from participants with more than 5 LTPA assessments within an 8-year period to establish the latent trajectories of LTPA. We acknowledge that there might have been bias in the information collected through questionnaires, and this issue should be taken into account when interpreting our results.

In conclusion, using data from a large cohort with long-term follow-up, we demonstrated that a medium/stable or increasing LTPA trajectory was independently associated with a lower risk of all-cause mortality in a general population. The association was independent of mean levels of LTPA during the study period. Our findings suggest that promotion of maintaining a stable LTPA level in a general population is benefit for public health and survival.
