# Supplementary material for: Associations of Long-Term Physical Activity Trajectories With All-Cause Mortality in a General Population
Source: Int J Public Health. 2023 Jan 16;68:1605332. doi: 10.3389/ijph.2023.1605332 (PMC9884672; doi:10.3389/ijph.2023.1605332)
Supplement: Supplementary file 1 [file Table1.DOCX]

|  | | | | | |
| --- | --- | --- | --- | --- | --- |
| Supplementary Table 1. Model building process of physical activity trajectory | | | | | |
| Assigned group of trajectory | Exact group of trajectory | BIC | Log Bayes factor  $\boldsymbol{\approx}$2x[ΔBIC] | Minimal % of group  Membership probabilities | Trajectory plot |
| 2 | 2 | -508696.7 |  | 12.9 | 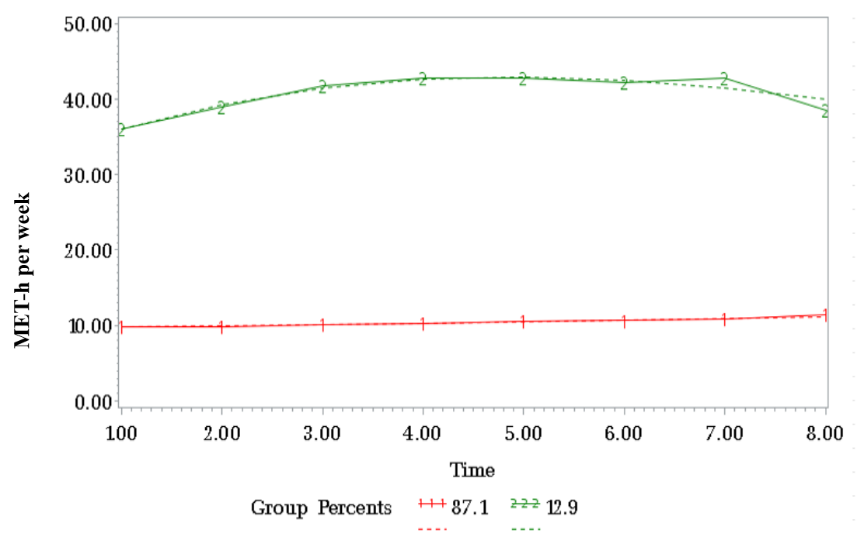 |
| 3 | 3 | -505004.8 | 7383.8 | 2.8 | 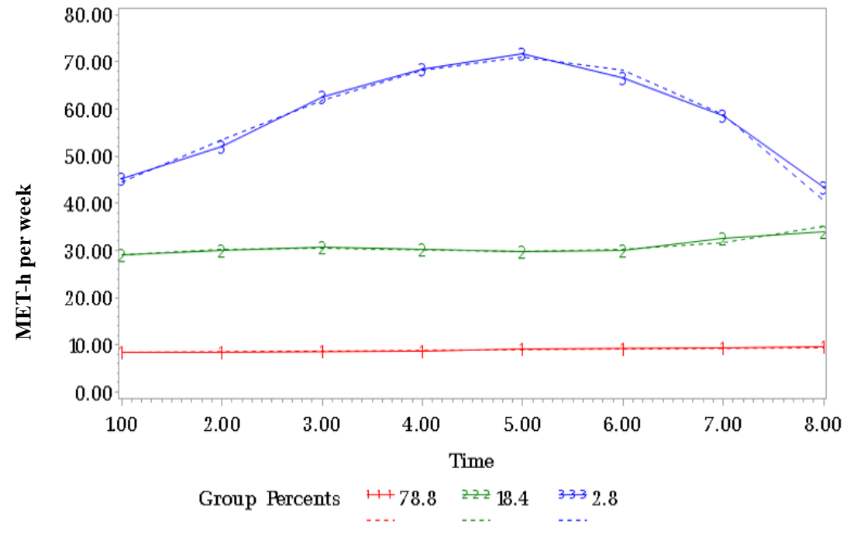 |
| 4 | 4 | -502422.7 | 5164.2 | 2.4 | 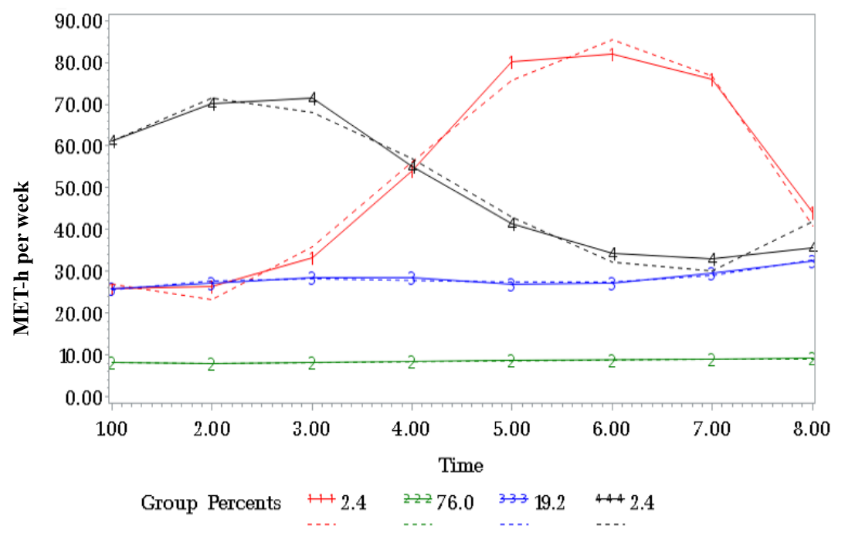 |
| 5 | 5 | -500280.6 | 4284.2 | 1.5 | 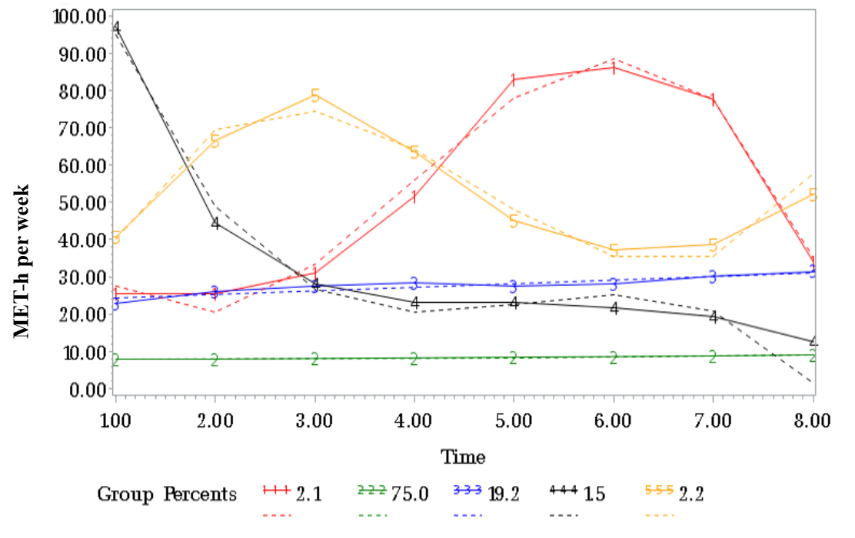 |
| 6 | 6 | -499513.7 | 1533.8 | 0.6 | 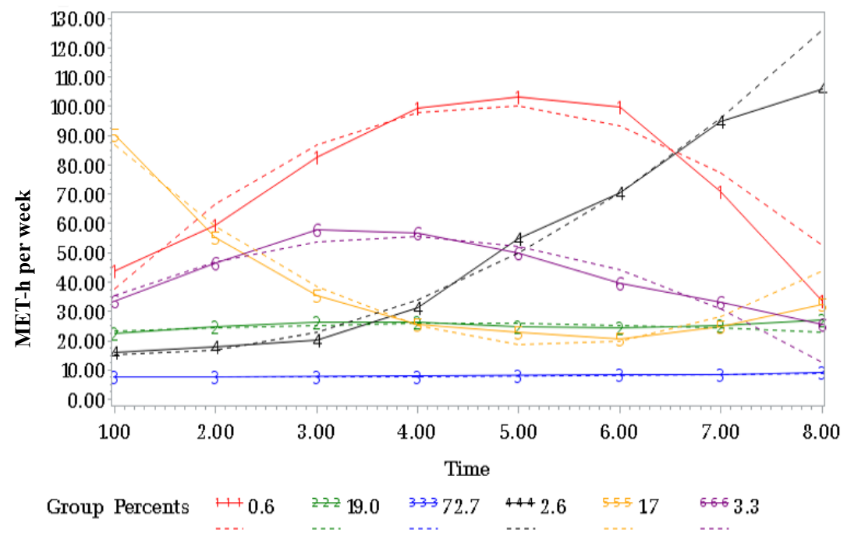 |
| Estimate of the log Bayes Factor defined by the following formula: Log Bayes factor ≈2x[ΔBIC] = 2x[complex model- simpler model] (Jones, Nagin, & Roeder, 2001, <https://www.andrew.cmu.edu/user/bjones/pdf/ref1.pdf>, accessed Dec 20, 2022). Among all the 20211 study participants, 11766 had 5 check-ups, 5926 had 6 check-ups, 2665 had 7 check-ups, and 854 had 8 check-ups within an 8-year period between 1996 and 2014. | | | | | |

| Supplementary Table 2. Associations of baseline characteristics and physical activity levels with all-cause mortality | | | | | |
| --- | --- | --- | --- | --- | --- |
|  | Univariate analysis | |  | Multivariate analysis^a^ | |
|  | HR (95% CI) | P |  | HR (95% CI) | P |
| Age, year | 1.11 (1.10-1.11) | <0.001 |  | 1.11 (1.09-1.14) | <0.001 |
| Sex (male vs. female) | 1.45 (1.27-1.65) | <0.001 |  | 1.03 (0.62-1.72) | 0.900 |
| Body mass index, kg/m^2^ | 1.08 (1.06-1.10) | <0.001 |  | 0.96 (0.90-1.02) | 0.208 |
| Systolic blood pressure, mm Hg | 1.03 (1.03-1.04) | <0.001 |  | 1.02 (1.00-1.03) | 0.007 |
| Smoking (yes vs. no) | 1.70 (1.47-1.97) | <0.001 |  | 2.27 (1.40-3.67) | 0.001 |
| History of CVD (yes vs. no) | 4.51 (3.53-5.77) | <0.001 |  | 1.30 (0.63-2.69) | 0.473 |
| Fasting plasma glucose, mmol/L | 1.01 (1.01-1.02) | <0.001 |  | 1.01 (1.00-1.01) | 0.213 |
| eGFR, ml/min/1.73m^2^ | 0.95 (0.95-0.96) | <0.001 |  | 0.99 (0.98-1.01) | 0.368 |
| Total cholesterol, mmol/L | 1.01 (1.01-1.01) | <0.001 |  | 1.00 (0.99-1.00) | 0.256 |
| Household income per year (NT dollar) |  |  |  |  |  |
| 0-<400 thousands | Reference |  |  | Reference |  |
| 400-<800 thousands | 0.52 (0.31-0.88) | 0.014 |  | 1.36 (0.78-2.37) | 0.284 |
| 800-<1200 thousands | 0.53 (0.31-0.89) | 0.017 |  | 1.48 (0.83-2.62) | 0.186 |
| 1200-<2000 thousands | 0.36 (0.18-0.69) | 0.002 |  | 0.99 (0.49-2.00) | 0.967 |
| >2000 thousands | 0.57 (0.26-1.25) | 0.158 |  | 1.19 (0.50-2.85) | 0.696 |
| Physical activity, 20 percentile increase | 1.29 (1.23-1.36) | <0.001 |  | 0.89 (0.77-1.02) | 0.094 |
| ^a^Adjusted for age, sex, body mass index, systolic blood pressure, smoking, history of cardiovascular disease (CVD), fasting plasma glucose, estimated glomerular filtration rate (eGFR), total cholesterol, social economic status, and physical activity. | | | | | |
